# Antifungal activity of *Gracilaria corticata* methanol extract against *Trichophyton mentagrophytes*, *Microsporum canis*, and *Microsporum gypseum* on rat dermatophytosis models

**DOI:** 10.18502/CMM.2023.150672

**Published:** 2023-03

**Authors:** Asiyeh Shojaee, Alireza Jahandideh, Ayatollah Nasrollahi Omran, Nakisa Sohrabi Haghdoost, Mehrzad Khosravi

**Affiliations:** 1 Division of Physiology, Department of Basic Sciences, Faculty of Veterinary Medicine, Ferdowsi University of Mashhad, Mashhad, Iran; 2 Department of Veterinary Surgery, Science and Research Branch, Islamic Azad University, Tehran, Iran; 3 Department of Mycology, Faculty of Medical sciences, Tonekabon Branch, Islamic Azad University, Tonekabon, Iran; 4 Department of Pathobiology, School of Veterinary, Science and Research Branch, Islamic Azad University, Tehran, Iran

**Keywords:** Algae, Animal model, Antifungal activity, Dermatophytosis, *Gracilaria corticata*

## Abstract

**Background and Purpose:**

Dermatophytosis is one of the most prevalent zoonotic diseases. Increased resistance of dermatophytosis-causing pathogens against antidermatophytic agents highlights the need for alternative medicine with higher efficiency and lower side effects. In the present study, the *in vitro* antifungal activities of different concentrations of *Gracilaria corticata* methanol extract against *Trichophyton mentagrophytes*, *Microsporum canis*, and *Microsporum gypseum* were assessed and their efficacy was evaluated in rat dermatophytosis models.

**Materials and Methods:**

The broth microdilution and well diffusion methods were used to determine the *in vitro* antidermatophytic activity. The *in vivo* study was carried out using 40 dermatophytosis-infected adults male Wistar rats. The animals were divided into 4 groups (5% and 10% *G. corticata* ointment, terbinafine, and Vaseline) and treated with ointment until complete recovery. The percentage of wound closure was calculated for each group.

**Results:**

The results revealed that *G. corticata* methanol extract was effective to varying extents against the tested dermatophytes. The highest inhibitory activity of *G. corticata* was found against *T. mentagrophytes* with minimum inhibitory concentration and minimum fungicidal concentration values of 4 and 9 µg mL^-1^, respectively. The *in vivo* experiment revealed that 10% *G. corticata* ointment significantly accelerated skin lesions reduction and completely cured *M. gypseum*, *T. mentagrophytes*, and *M. canis* infections after 19, 25, and 38 days, respectively.

**Conclusion:**

The methanol extract of *G. corticata* exhibited significant antifungal activity *in vitro* and *in vivo*, suggesting that it could be used as an alternative to antidermatophytic therapy in a dose-dependent manner.

## Introduction

Dermatophytoses are superficial fungal infections caused by dermatophytes that invade and feed on keratinized tissues, like the epidermis, hair, and nails, causing an infection. Incidence rate of these pathogens is higher in hot and humid climates areas with poor hygienic conditions and over-populated regions [ 1 , 2 ] . 

Dermatophytosis is caused by a group of fungi known as dermatophytes belonging to the *Trichophyton*, *Microsporum*, or *Epidermophyton* genera [ 3 - 6 ]. *Microsporum canis* is a zoophilic dermatophyte and is the most common dermatophyte pathogen in cats and dogs [ 7 , 8 ]. It is a geophilic dermatophyte that typically accompanies inflammatory reactions [ 9 ].

Most members of the *Trichophyton* genus are anthropomorphic. In addition, dermatophytosis caused by *Trichophyton mentagrophytes*, which is a zoophilic fungus, mainly causes disease in rodents and rabbits, compared to cats, dogs, and other animals [ 9 ]. Usage of topical and systemic drugs to treat dermatophytosis is often associated with fungal resistance, high cost, and side effects. Therefore, it is necessary to conduct studies on effective and safe alternative medicine based on plant compounds [ 10 , 11 ].

Seaweeds belonging to a group of plants known as algae have attracted a lot of attention in recent years. These plants are used for many applications, such as pharmaceuticals, cosmetics, functional foods, and food packaging [ 12 ]. Marine algae are classified as red algae (Rhodophyta), brown algae (Phaeophyta), and green algae (Chlorophyta) [ 13 ]. Among red algae, the genus Gracilaria is a source of important bioactive metabolites and exhibits many biological activities, including antioxidant, anti-inflammatory, antimicrobial, anti-ulcer, anti-cancer, anti-lipid, and anti-diabetic activities [ 14 ]. Wide distribution of *G. corticata* in the coastal areas of Iran requires more research to be conducted about it from different perspectives [ 15 ].

Due to the higher prevalence rate of common types of dermatophytosis and the growing popularity of keeping pets, this study, aimed to investigate the *in vitro* antifungal effects of *G. corticata* methanol extract against *T. mentagrophytes*, *M. canis*, and *M. gypseum*. Since *in vivo* studies using animal models are pivotal for testing the efficacy of antifungal compounds, their efficacy was also evaluated in rat dermatophytosis models [ 7 , 16 ].

## Materials and Methods

### Dermatophytes

*Trichophyton mentagrophytes* (PTCC5054), *M. canis* (PTCC5069), and *M. gypseum* (PTCC5057) that were used in this study were purchased from the Persian Type Culture Collection (PTCC, Tehran, Iran).

### Antifungal agent

In this study, *G. corticata* was collected from the Chabahar coast in Baluchistan province, Iran. The algae were washed; afterward, the cleaned algae were air-dried and powdered. The powdered sample was extracted by soaking it in methanol according to the method proposed by Saideni et al. [ 17 ]. Briefly, the extract was filtered and centrifuged, then the supernatant was concentrated and dissolved in methanol. Afterward, the cconcentrated extract was filtered and stored at 4 °C for further use and analysis.

### Animal

In total, 40 adult male Wistar rats (200-250 g) were obtained from the laboratory animal house of the Faculty of Veterinary Medicine of the Science and Research Branch, Islamic Azad University, Tehran, Iran. The rats were housed in a room under controlled temperature (22±2 °C) and humidity (50 °C±10%) with a 12/12 h light/dark cycle and free access to food and water.

All methods were carried out following relevant guidelines and regulations. Rats were humanely treated following the Animal Research: Reporting of *in Vivo* experiments guidelines for animal care [ 18 ]. Moreover, all *in vivo* experimental procedures were approved by the Research Ethics Committee of the Science and Research Branch, Islamic Azad University (IR.IAU.SRB.REC.1399.196).

### Preparation of fungal suspension

*Trichophyton mentagrophytes*, *M. canis*, and *M. gypseum* were used in this study. Each sample was cultured on Sabouraud Dextrose Agar (Ibresco, Iran) and the plates were incubated at 30 °C for up to two weeks. Afterward, a suspension was prepared from the colony of dermatophytes using normal saline solution and Tween 80 with a final concentration of 1×10^6^ spores mL^-1^.

### In vitro assays

#### Antimicrobial susceptibility testing

The minimum inhibitory concentration (MIC) of *G. corticata* and terbinafine against the tested dermatophytes was determined using the broth microdilution method recommended by the Clinical and Laboratory Standards Institute M38-A2 method [ 19 ]. Serial dilutions of antifungal agents starting at 70 µg ml^-1^ of *G. corticata* were prepared and compared with the reference antifungal drug, which was terbinafine. Concentrations of *G. corticata* and terbinafine were within the ranges of 78-0.125 and 4-0.008 µg ml^-1^, respectively [ 20 ].

### Well diffusion method

The agar well diffusion method was used to assess the inhibitory activity of *G. corticata* methanol extract. Effectiveness of the active solution was calculated by measuring the inhibition zone around three wells. Moreover, the percentage of dermatophyte growth inhibition was calculated as follows [ 21 ]:

FI (%)=(IR/GR)×100

FI: fungal inhibition, IR: inhibition radius, GR: growth radius

### In vivo assays

#### Dermatophytosis model

The rats were anesthetized by intraperitoneal injection of ketamine/xylazine. Backs of the animals were shaved and their skins were gently rubbed with sterile fine sandpaper to make them susceptible to infection.

Afterward, their skins were inoculated with fungal suspension [ 16 ]. After 4 days of inoculation, the samples were collected and cultured. Moreover, to confirm the infection, direct microscopic examination was performed in 8-10 days.

### Experimental group

On day 10 after the infection, the rats were randomly divided into four groups, and the wound areas were covered with each of the assigned treatments once a day. It should be noted that this procedure continued until complete recovery was achieved. The study groups included 1) negative control: inoculated rats that were treated with Vaseline ointment; 2) positive control: inoculated rats that were treated with terbinafine ointment; 3) inoculated rats that were treated with 5% *G. corticata* ointment; and 4) inoculated rats that were treated with 10% *G. corticata* ointment. *Gracilaria corticata* ointment was prepared by mixing 5 and 10 ml of *G. corticata* methanol extract (MIC value) with 100 g of Vaseline ointment.

### Wound surface area measurement

To evaluate the therapeutic efficacy of *G. corticata* ointment, the wound area was assessed daily and measured manually using a transparent grid throughout the treatment period to determine the recovery rate of the infected site. The percentage of wound closure was calculated based on the following equation: wound closure (%)=(A_0_–A_n_/A_0_)×100 

A_0_ represents the wound area at day 0 and A_n_ refers to the wound surface area at a different time point [ 22 ]. 

### Statistical analysis

The results were expressed as mean values of three independent replicates. The results were analyzed using analysis of variance followed by Tukey's honestly significant difference. *P* values of less than 0.05 were considered statistically signiﬁcant. 

## Results

### In vitro antidermatophytic effect of Gracilaria corticata

The *in vitro* antifungal effects of *G. corticata* methanol extract was evaluated against *T. mentagrophytes*, *M. canis*, and *M. gypseum*, and their MIC, Minimum fungicidal concentration (MFC), and percentage of growth inhibition were determined. It was found that all tested dermatophytes were highly sensitive to *G. corticata* methanol extract (Table 1).

**Table 1:** *In vitro* antifungal activity of antidermatophytic agents

	Gracilaria corticata			Terbinafine	
Tested fungi	MIC	MFC	WD FI (%)	MIC	MFC
*Trichophyton mentagrophytes *	4	9	52 ± 0.056^a^	0.2	4
*Microsporum canis*	9	19	34 ± 0.028 ^b^	0.1	0.2
*Microsporum gypseum*	39	78	28 ± 0.028^c^	4	9

MIC: Minimum inhibitory concentration; MFC: Minimum fungicidal concentrations. WD: Well diffusion methods; Different superscripts within the same row indicate that the means differ significantly (*P < 0.05*).

In the microdilution assay, the MIC and MFC values were within the range of 4-78 µg mL^-1^. Moreover, it was revealed that *G. corticata* methanol extract showed the highest inhibitory activity against *T. mentagrophytes* with a MIC value of 4 µg mL^-1^. 

Table 1 also represents the antidermatophytic potential of *G. corticata* methanol extract, compared to synthetic anti-dermatophyte drugs. The MIC and MFC values of terbinafine ranged from 0.1 to 9, which are much less than the MIC of *G. corticata* methanol extract. The fungus *T. mentagrophytes* was found to be more sensitive than *M. canis* and *M. gypseum*. The well diffusion method showed that the greatest growth inhibition rate of the extract was observed against *T. mentagrophytes* (52±0.056%), followed by *M. canis* (34±0.028%) and *M. gypseum* (28±0.028) at their MIC concentrations.

### In vivo antidermatophytic activity of Gracilaria corticata

To evaluate the efficacy of *G. corticata* ointment, different degrees of skin lesions among various treatment groups were assessed and recorded throughout the study period. All rats infected with dermatophyte showed signs of inflammation on the third day. Severity of lesions reached the highest level almost on day 10, which indicated the growth of dermatophyte fungus in the infected areas.

**Figure 1:**
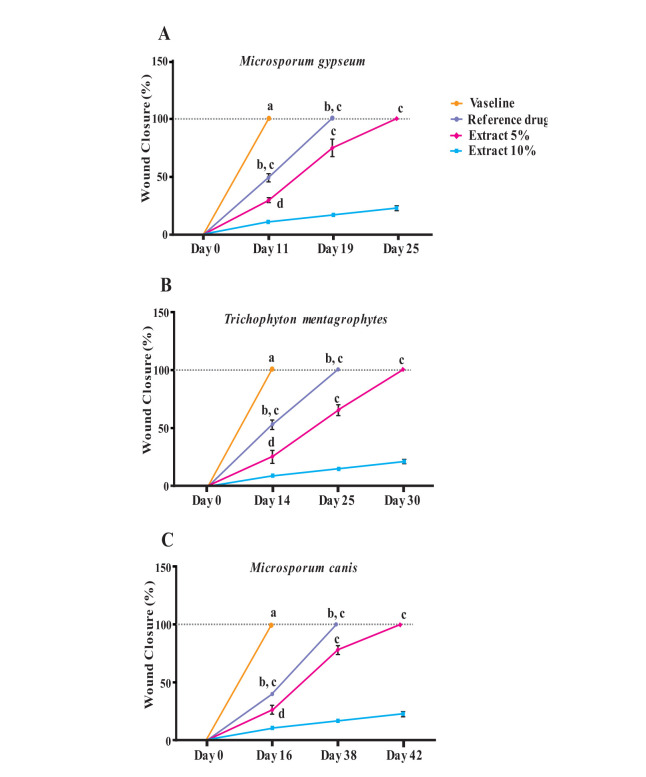
Time course of recovery of dermatophytes infection in animal groups treated with terbinafine as the positive control, tested ointments (5 and 10 % *Gracilaria corticata* methanolic extract), and Vaseline as the negative control.

Visual examination of skin lesions after treatment showed improvement in the clinical signs of infection, while patches of hair loss and scaly skin were observed in the negative control group. The first signs of recovery were observed in animals infected with *M. gypseum* followed by animals infected with *T. mentagrophytes* and *M. canis* (Figure 1). Both concentrations of *G. corticata* led to a significant decrease in lesion size and hair growth in the infected sites, compared to the negative control. However, 10% *G. corticata* ointment significantly accelerated skin wound healing.

In the *M. gypseum*-infected rats (Figure 1A), 100% wound healing was achieved on days 19 and 25 in 10% and 5% *G. corticata* groups, respectively, compared to terbinafine as a positive control on day 11. It was revealed that the wound closure rate on day 11 in the 10% *G. corticata* group was significantly lower, compared to the terbinafine group (50% vs. 100%, *P*<0.0001), while it was significantly higher than 5% *G. corticata* (50% vs. 25 %, *P*<0.01). It was also indicated that 5% *G. corticata* ointment showed higher therapeutic eﬀects on the dermatophyte infection, compared to Vaseline (*P*<0.0001).

As can be seen (Figure 1B), in *T. mentagrophytes*-infected rats, wound healing reached 100% on day 14 in rats treated with terbinafine. It was also indicated that complete recovery from the infection was observed 25 days after the treatment in rats treated with 10% *G. corticata* topical ointment, whereas those treated with 5% extract ointment were completely cured on the 30th day of treatment. 

In the case of *M. canis*-infected rats (Figure 1C), no sign of lesion was observed in the terbinafine treatment group on day 16, while 100% recovery was observed on the 38^th^ day of treatment in the 10% *G. corticata* topical ointment group and 5% *G. corticata* topical ointment group showed clear signs of recovery on 42^nd^ day. Antidermatophytic activities of different treatments had highly significant differences in terms of time and dose-dependence in the following order: terbinafine > 10% *G. corticata* > 5% *G. corticata* > Vaseline.

## Discussion

There have been several reports of antibiotic resistance in dermatophyte pathogens which has made the commercialization of alternative antidermatophytic agents a necessity. The herbal-based medicines have attracted great attention as favorable candidates for antidermatophytic therapy [ 23 ]. The present study investigated the *in vitro* and *in vivo* antidermatophytic activities of *G. corticata* methanol extract. In this study, it was found that *G. corticata* exhibited strong *in vitro* antidermatophytic activities against *T. mentagrophytes*, *M. canis*, and *M. gypseum*. 

It was also demonstrated that the therapeutic effects of *G. corticata* ointments in the rat model are dose- and time-dependent. To our knowledge, this is the first report of the antifungal activity of *G. corticata* methanol extract against dermatophyte fungi. There have been some studies on the antifungal effects of seaweed extracts [ 24 - 26 ]; however, there are not many published papers regarding their antidermatophytic effects. 

It has been reported that the MIC values of the *Bifurcaria bifurcata* and brown algae against *T. mentagrophytes*, *M. canis*, and *M. gypseum* were 100, 400, and 800 µg mL^-1^, respectively [ 27 ]. But these seaweed extracts were less effective in comparison to *G. corticata* methanol extract against *T. mentagrophytes*, *M. canis*, and *M. gypseum* in the present study with MIC values of 4, 19, and 78 µg mL^-1^, respectively. 

However, it should be noted that comparison was diﬃcult as the used seaweeds were not the same. Nevertheless, *T. mentagrophytes* showed the highest sensitivity to both seaweeds followed by *M. canis* and *M. gypseum*. The same results have been reported for the antifungal activity of *Lobophora variegata* and brown alga against *T. mentagrophytes*. However, the methanol extract of *L. variegata* exhibited higher antifungal activity with an inhibition zone diameter (IZD) of 11.42±0.002 mm, 62.64% at 100 μg mL^-1^, compared to other solvent extracts [ 28 ].

Another previous study has investigated the antifungal efficacy of methanolic extract from marine brown seaweed, *Colopomenia peregrine*, at 30 µg ml^-1^ concentration against *M. gypseum* and reported an IZD value of 12 mm [ 29 ]. In another study performed on the antifungal efficiency of various solvent extracts of red algae, Acanthaphora spicifera, it was revealed that the methanol extracts showed maximum antifungal activity and the zone of inhibition was 12 mm against *M. gypseum* at a concentration of 50 mg/ml^-1^ [ 30 ].

In contrast to the results of the antidermatophytic activity of selected algae against the tested dermatophytes, the green, brown, and red algae obtained from Brazil showed strong activity with low MIC values ranging from 0.03 to 16.00 µg ml^-1^. *Microsporum canis* showed a MIC value of 0.03 µg ml^-1^ with an IZD value of 10-25 mm. The ethanol extract was found to be the most effective growth inhibitor of the fungi [ 31 ].

The terpenoids, tannins, and phenolic compounds were present in the different extracts of *G. corticata* isolated from the Persian Gulf [ 32 ]. It has been reported that the possible antimicrobial mechanism of the seaweeds is related to their phlorotannins extracts. This mechanism acts by affecting the fungal cell wall and membrane composition as well as the mitochondrial function [ 33 ]. A series of bromophenols isolated from red alga showed MIC values of 1.56, 12.5, 25, 50, and >100 µg ml^-1^ against *T. mentagrophytes* [ 34 ]. 

Effects of anti-fungal activity greatly depend upon the factors influencing the geographical region, sampling period, species variation, environmental variations and climatic conditions, extraction method, and chemical composition of the seaweed [ 35 ]. Considering the potential *in vitro* activity of algae, the efﬁcacy of its *in vivo* application against induced dermatophytosis in the rat model was also investigated. There are several clinical reports on *in vitro* anti-dermatophytic efficacy of natural sources [ 35 - 40 ], while just some limited investigations have been performed on the *in vivo* effects of algae. 

In contrast to *in vitro* results, *in vivo* results showed that *M. gypseum* was the most sensitive species to the methanol extracts of *G. corticata*, while *M. canis* was less effective. This finding might be attributed to the immune response of animals to dermatophytes which leads to self-healing. In this way, the average area of skin lesions of rats infected by *M. gypseum*, *T. mentagrophytes*, and *M. canis* in negative control was slightly decreased in a time-dependent manner.

Results of the present study are in agreement with those obtained by other investigators which have shown the *in vivo* anti-dermatophytic activity of algae against dermatophytes. The ethanol extract of the algae showed promising antifungal activity against *T. mentagrophytes* with a MIC value of 62.5 μg mL^-1^. *In vivo* evaluation of the ethyl acetate soluble fraction of the ethanol extract (MIC: 15.8 μg mL^-1^) in a multi-infection fungal model in Swiss albino mice provided 60% protection at 50 mg/kg p.o. with a reduced CFU [ 41 ].

Yu-Xia reported *in vivo* antifungal activity of different concentrations (0.1%, 1%, and 5%) of chitooligosaccharides (COS) derived from chitosan, against *T. rubrum* using a guinea pig model. In the aforementioned study, the 5% COS group showed a significant reduction in skin lesions, compared to the positive control group that received 1% fluconazole. Similar to the results of the present study, skin lesion reduction revealed the dose-dependent therapeutic effect of COS [ 42 ]. Several reports have shown that chitosan, which is derived from chitin and is present in algae, can induce morphological change in the fungal hyphae and acts as a chelating agent to limit nutrients for fungal growth [ 43 , 44 ].

It is well known that the presence of functional bioactive compounds in seaweed extract is responsible for its high anti-dermatophytic activity. Results of a study conducted by Radhika *et al*. [ 45 ] have reported the effectiveness of green algae, Chlorella vulgaris, and ointment on *M. canis*-infected rats after the production of a thermal lesion on its back. They showed that *C. vulgaris*, which is rich in phenolic compounds, was effective and also revealed the normal histological situation of the skin tissues of rats after the treatment [ 46 ]. In the present study, it seemed that the high amount of total phenolics in *G. corticata* might be the reason for its high anti-dermatophytic activity [ 45 , 47 ].

It has been reported that lectins isolated from some red algae exhibited analgesic, anti-microbial, anti-ulcerogenic, and anti-inﬂammatory effects [ 48 ]. Topical treatment of lectin isolated from *Bryothamnion seaforthii* on induced skin wounds in mice showed a significant healing potential [ 49 ]. Although the used wound-induced model organism was not the same as the one in the present study, the results of both studies showed that the topical application of the investigated algae may increase their penetration into the skin and lead to a faster wound-healing process.

## Conclusion

The results obtained in the present study provide scientiﬁc validation for the anti-dermatophytic activity of *G. corticata* methanol extract at low concentrations. Moreover, the findings also indicated its *in vivo* efficacy in rat models infected with *T. mentagrophytes*, *M. canis*, and *M. gypseum*.

The collected data confirmed the potential applications of algae extracts against tested dermatophytes which can motivate future studies on its active ingredients, mechanisms of action, and randomized clinical trials. As various algal species widely grow in different tropical regions of the world, they may be recommended as natural resources in the treatment of dermatophyte infections as well as an alternative to a synthetic drug for topical applications in a dose-dependent manner.

## Acknowledgments

Not declared. 

## Authors’ contribution

The authors declare that all listed authors have made equal contributions to the conceptualization, formal analysis, methodology, review preparation, and edition of the present research. All authors have read and approved the submitted final manuscript.

## Conflicts of interest

The author states that there is no conflict of interest.

## Financial disclosure

No financial interests related to the material of this manuscript have been declared.
